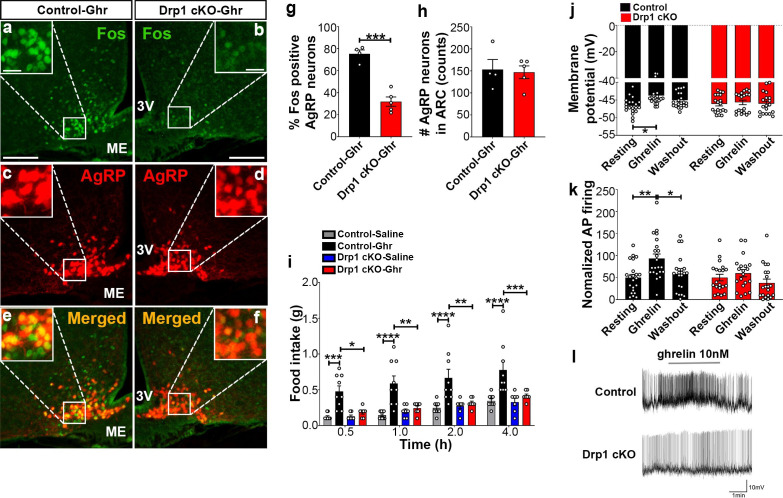# Correction: Drp1 is required for AgRP neuronal activity and feeding

**DOI:** 10.7554/eLife.80570

**Published:** 2022-06-28

**Authors:** Sungho Jin, Nal Ae Yoon, Zhong-Wu Liu, Jae Eun Song, Tamas L Horvath, Jung Dae Kim, Sabrina Diano

**Keywords:** Mouse

Jin S, Yoon NA, Liu Z-W, Song JE, Horvath TM, Kim JD, Diano S. 2021. Drp1 is required for AgRP neuronal activity and feeding. *eLife* **10**:e64351. doi: 10.7554/eLife.64351.

Published 9 March 2021

We discovered that the line used to indicate the time when ghrelin 10nM was added during the electrophysiological recordings of the two representative AgRP neurons in Fig. 7l of the original version of our manuscript got misplaced in its final version. The line should be shifted toward the left to accurately point the time of ghrelin addition during the electrophysiological recordings. The article has been corrected accordingly.

The revised Figure 7 (panel l) is shown here:

**Figure 1:**
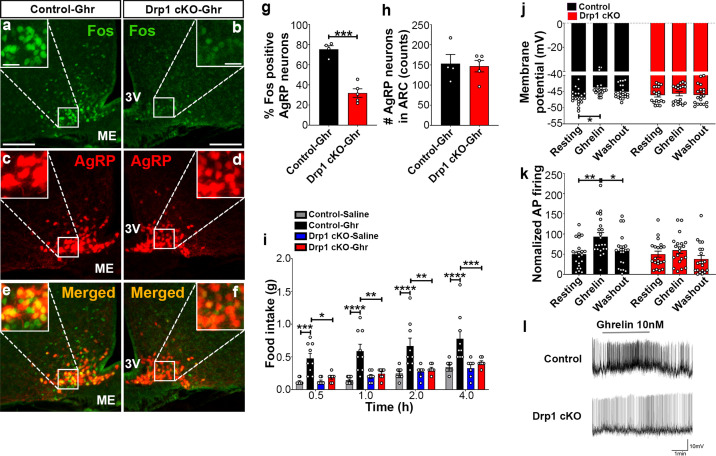


The current published Figure 7 is also shown for reference:

**Figure 2:**